# Special Issue—“Isolation, Structure Elucidation and Biological Activity of Natural Products”

**DOI:** 10.3390/molecules28145392

**Published:** 2023-07-14

**Authors:** Jacqueline Aparecida Takahashi

**Affiliations:** Exact Sciences Institute, Universidade Federal de Minas Gerais (UFMG), Av. Antonio Carlos, 6627, Belo Horizonte 31270-901, Brazil; takahashi.ufmg@gmail.com

This Special Issue of *Molecules* gathers fourteen research studies and three review papers covering developments in the scope of the isolation, structure elucidation and biological activity of natural products. Plants are undoubtedly the most well-known sources of bioactive natural products, since plants have been used medicinally by traditional communities for centuries, defining their specific benefits to human health. In this Special Issue, the plants addressed are *Bauhinia forficata*, *Placolobium vietnamense*, *Tamarix chinensis*, *Peperomia obtusifolia*, *Cymbidium ensifolium*, *Dendrobium delacourii*, *Turnera subulata*, *Eruca sativa*, *Miconia chamissois* and *Persea americana*, with the aim of achieving the identification of their chemical compositions, the prospection of biological activities, the standardization of extracts and the use of agro-industrial residues. Some studies involved hyphenated techniques such as UPLC-IT-MS^n^ [1], UHPLC-ESI-QTOF [2], UHPLC/UV/MS/MS [3], PSMS [4] and UPLC–MS/MS [5], which are very useful tools in research on natural products.

*B. forficata* is a tropical species popularly used in Brazil to treat type II diabetes, rheumatism, local pain, uric acid and uterine problems. Jung et al (2022) carried out a comparative study between locally collected leaves of this species and commercial samples [6]. Using LC-HRMS, a very useful hyphenated analytical technique, the authors were able to identify the presence of flavonoids, phenolic acids and other phenolic volatile compounds, including flavonoid O-glycosides in the plant, and these metabolites were related to the pharmacological actions reported for *B. forficata.* The plant inhibited the α-amylase enzyme, and the results converged to reinforce the biological and pharmacological potential of *B. forficata* as a hypoglycemiant agent [6]. Studies of this type are of great value, especially considering that *B. forficata* is an easily accessible plant for the population and is already commercialized.

*P. vietnamense,* known in Vietnam as “Rang Rang”, is also distributed throughout the world’s tropical regions and is used as a folk medicine to treat snakebites, debility and to increase strength after childbirth. Stems of *P. vietnamense* were studied by Do, Huynh, and Sichaem (2022), who successfully isolated eight natural constituents of this species, including a new isoflavone derivative and three new benzyl derivatives, together with four known compounds of the pyranoisoflavone type [7]. The authors conducted a cytotoxic evaluation of the effects of the aforementioned natural products on a human hepatocellular carcinoma (Hep G2) cell line, determined the cytotoxic effects and measured the production of nitric oxide (NO) by RAW 264.7 cells. Among the compounds tested, placovinone A exhibited the most significant cytotoxicity toward the Hep G2 cell line and also strongly inhibited LPS-induced NO production [7]. The authors concluded that placovinone A is a promising lead for discovering potential anticancer and anti-inflammatory agents.

The work of Jiao et al. (2022), conducted with the Chinese plant *T. chinensis*, which is used to treat rheumatoid arthritis, measles and measles complicated with pneumonia, is an excellent example of the success of activity-guided extraction, isolation and purification targeting a specific class of compounds [1]. In this case, the authors were focused on the bioactive polysaccharides present in *T*. *chinensis*. Flavonoids, triterpenoids, organic acids and volatile oils with anti-inflammatory, bacteriostatic, antioxidant and hepatoprotective effects have already been described from this plant. An activity-guided approach, followed by careful spectroscopic study (HPGPC-ELSD, UPLC-IT-MS^n^ and NMR analysis), led to the isolation and identification of two novel natural flavonoid-substituted polysaccharides. Both polysaccharides were substituted by quercetin and exhibited anticomplement activities in vitro. Since the authors were focusing on inflammatory responses in viral pneumonia, antioxidant activities were also determined for the polysaccharides. The authors indicated that molecules containing both flavonoids and polysaccharides have advantageous drug delivery. Structure–activity studies showed that multiple monosaccharides also contribute to the anticomplement activity of *T. chinensis* flavonoid-substituted polysaccharides [1].

*P. obtusifolia* is an ornamental plant species that is widely distributed in tropical areas. Plants from the *Peperomia* genus have been traditionally used to treat asthma, gastric ulcers, bacterial infection, pain and inflammation. Ware et al. (2022) isolated two new phenolic compounds from *P. obtusifolia* (peperomic ester and peperoside), along with other five known metabolites that were screened for their anthelmintic (*Caenorhabditis elegans*), antifungal (*Botrytis cinerea*, *Septoria tritici* and *Phytophthora infestans*) and antibacterial (*Bacillus subtilis*, *Aliivibrio fisheri*) activities, as well as for their cytotoxicity against human prostate (PC-3) and colorectal (HT-29) cancer cell lines [8]. One of the metabolites tested, peperobtusin A, strongly affected the viability and growth of PC-3 cells in MTT and a CV fast-screening assay and was moderately active against the HT-29 cell line [8]. The results are an indicative that ornamental plants can also be sources of bioactive metabolites.

The roots of *Cymbidium ensifolium*, another ornamental plant known as “nang kham” or “chulan” in Thailand, are used in traditional Thai medicine to alleviate liver dysfunction and nephropathy. The aerial parts of *C. ensifolium* were studied by Jimoh et al (2022), who isolated three novel dihydrophenanthrene derivatives (cymensifins A, B and C), along with two known compounds, cypripedin and gigantol [9]. The chemical structures of the dihydrophenanthrene derivatives were elucidated, mainly via HMBC and NOESY correlations. Their activity against human lung cancer H460, breast cancer MCF7 and colon cancer CaCo_2_ cells were evaluated in cell viability assays and for apoptosis/necrosis. The most promising anticancer compound was cymensifin A, which was found to be active against different cancer cells with higher safety profiles compared with the control, cisplatin [9].

Another orchid, *Dendrobium delacourii*, named “Ueang Dok Ma Kham” in Thailand, was studied by Thant et al. (2022) [10]. They isolated 11 compounds and identified them as hircinol, ephemeranthoquinone, densifloral B, moscatin, 4,9-dimethoxy-2,5-phenanthrenediol, gigantol, batatasin III, lusianthridin, 4,4′,7,7′-tetrahydroxy-2,2′-dimethoxy-9,9′,10,10′-tetrahydro-1,1′-biphenanthrene, phoyunnanin E and phoyunnanin C. Dimeric phenanthrene derivatives presented stronger α-glucosidase inhibitory activity than the monomers. A kinetic study indicated the active metabolites to be non-competitive enzyme inhibitors. The authors argued the benefits of non-competitive inhibitors over competitive inhibitors. Regarding anti-adipogenic action, ephemeranthoquinone and densifloral B showed the highest levels of activity; the latter restrained adipocyte differentiation in 3T3-L1 cells in a dose-dependent manner [10]. The authors suggested that densifloral B might inhibit adipocyte differentiation via the suppression of the Akt-mediating GSK3β and AMPK–ACC signals. These results are of great interest since diabetes and obesity are major problems worldwide.

Extracts from flowers of the tropical plant *Turnera subulata* were studied by Luz et al. (2022) [5]. Their in vitro immunomodulatory effects in RAW 264.7 macrophages stimulated via LPS were evaluated in the search for anti-inflammatory drugs with low side effects. Vitexin-2-*O*-rhamnoside was identified in the extracts via UPLC–MS/MS, as were methoxyisoflavones, pheophorbides, octadecatrienoic, stearidonic, ferulic acids and some amino acids. The results demonstrate the immunomodulatory effects of aqueous and hydroalcoholic extracts of *T. subulata* flowers and leaves through the inhibition of inflammatory TNF-α and IL-1β cytokine secretion [5]. Increases in anti-inflammatory IL-10 cytokine levels also supported the activity and corroborate the ethnopharmacological use of plants from the *Turnera* genus in folk medicine as anti-inflammatory remedies.

In addition to essentially medicinal and ornamental plants, one of the works published in this Special Issue focused on a plant used mainly as food, *Eruca sativa* (rocket). Based on works using animal models that associated rocket with biological activities, such as antihypertensive, nephroprotective and antidiabetic activities, Teixeira et al. (2022) hypothesized that this plant could have anti-hyperuricemic effects, combatting a causal factor of hypertension and diabetes [2]. Nine compounds were detected via UHPLC-ESI-QTOF: kaempferol-3-O-β-glucoside, kaempferol-3,4′-di-O-β-glucoside, kaempferol-3-O-(2-sinapoyl-β-glucoside)-4′-O-glucoside, glucosativin glucosinolate, glucoraphanin glucosinolate, leucine, tryptophan, angustione and erucamide. Kaempferol-3,4′-di-O-β-glucoside was elegantly characterized via NMR and quantified in the extract. the anti-hyperuricemic activities of the extracts were mainly related to uricosuric action [2]. Although other mechanisms are yet to be elucidated, the authors suggested that the results indicate the potential use of *E. sativa* in the treatment of hyperuricemia and its comorbidities.

Ferreira et al. (2022) raised an important issue in the study of medicinal plants, which is the standardization of extracts and the variations relating to seasonal variations. The authors focused on *Miconia chamissois*, known as “Folha de Bolo”, “Sabiazeira”, or “Pixirica” in Brazil, a plant associated with antimicrobial and antioxidant activities, the in vitro inhibition of the enzymes tyrosinase and alpha-amylase, the inhibition of MMP-2 and MMP-9 and cytotoxicity against human cervical cancer cell lines [3]. A standardized extract was formulated, and some major constituents, such as apigenin C-glycosides (vitexin/isovitexin), luteolin C-glycosides (orientin/isoorientin), miconioside B, matteucinol-7-O-β-apiofuranosyl (1 → 6)-β-glucopyranoside and farrerol, were identified (UHPLC-MS/MS). Ferreira et al. evaluated samples collected in the autumn, winter and spring, seasons with different environmental factors such as temperature, ultraviolet radiation, rain index and soil nutrients. No significant correlations between the meteorological data and the biological potential were observed, demonstrating that the species studied was well adapted to the different environmental conditions targeted in the study.

An important aspect linked to the use of plants, medicinal or not, was contextualized in the work of Silva et al. (2022), who studied the peels and seeds of avocado (*Persea americana*), which represent 30% of the fruit and are usually discarded as waste, generating environmental problems and the loss of bioactive compounds that remain in the biomass after processing [4]. The authors showed that avocado residues retain several nutrients such as minerals (Ca, Mg, Mn and Zn) and high amounts of essential fatty acids such as linoleic, palmitic and oleic acids. The chemical profile obtained via paper spray mass spectrometry (PSMS) showed fifty-five metabolites, including phenolic compounds, hydroxycinnamic acids, flavonoids and alkaloids, in the residues. The ethanol extract of the peels was the best acetylcholinesterase inhibitor, with no significant difference (*p* > 0.05) compared to the control, eserine. The seed extracts exhibited an in vivo neuroprotective effect against rotenone-induced damage in an in vivo *Drosophylla melanogaster* model [4]. This work contributed some interesting points to the scope of research on natural products, such as a preoccupation with sustainability and a circular economy, the choice of environmentally favorable solvents for extraction and the role of complementarity in vivo assays. Although toxicity, bioavailability and suitable formulations should be further investigated before using avocado residues in pharmacotherapy, this work showed the potential use of avocado residues as a bioresource in the development of low-cost drugs and functional foods with neuroprotective effects. 

Bacteria and fungi are also important producers of bioactive metabolites. The purification of a catechol-type siderophore from *Streptomyces tricolor* was studied with the aim of inducing recovery from iron-deficiency-induced anemia in in vivo rat model (Barakat et al., 2022) [11]. Siderophores are low-molecular-weight natural compounds secreted by microorganisms that act as iron chelators. Due to this characteristic, siderophores have useful therapeutic applications, especially in iron-overload diseases (hemosiderosis, β-thalassemia, hemochromatosis and accidental iron poisoning). Iron chelators are also useful in cancer therapy because cancer cells have higher requirements of iron when compared to healthy cells. Barakat et al. (2022) focused on applications related to the damages caused by iron-deficiency-induced anemia, showing that sidereophores improved weight gain and were effective during recovery from anemia [11]. The results led the authors to propose some hypotheses and created opportunities to delineate the detailed mechanism of the changes observed and elucidate iron pathways.

*Lacticaseibacillus rhamnosus* XN2, a bacteriocin-producing strain isolated from the naturally fermented yak yoghurt produced in Xining and Qinghai Provinces in China, was the target of the work of Wei et al. (2022) [12]. The source of the bacteria studied, yogurt from yak milk farmed in the Himalayan region, exemplifies the vastness of the area of natural products and the local value of the studies, as well as the global importance of the results in expanding knowledge in the area of biodiversity and ecosystem services. Bacteriocins from lactic acid bacteria are peptides secreted by some lactic acid bacteria with natural antimicrobial activities against other microorganisms, including food spoilage and pathogens. *L. rhamnosus* XN2 demonstrated antibacterial activity against *Bacillus subtilis*, *B. cereus*, *Micrococcus luteus*, *Brochothrix thermosphacta*, *Clostridium butyricum*, *S. aureus*, *Listeria innocua*, *L. monocytogenes* and *Escherichia coli.* Semi-purified, cell-free supernatants of *L. rhamnosus* XN2 showed bactericidal activity, probably due to the disruption of the sensitive bacteria membrane, as suggested by a flow cytometry analysis. The production of α-haemolysin and biofilm formation were observed for sub-lethal concentrations of the semi-purified material. Bacteriocin was further purified via reversed-phase high-performance liquid chromatography (RP-HPLC), and its amino acid sequence was determined to be Met-Lue-Lys-Lys-Phe-Ser-Thr-Ala-Tyr-Val [12]. The authors concluded that *L. rhamnosus* XN2 and its bacteriocin showed antagonistic activity at both cellular and quorum-sensing levels.

In relation to fungi, their secondary metabolites usually have diverse applications in the food, cosmetic, beverage and textile industries, and their biological activities enable their use in the development of new drugs. Some fungal pigments, such as azaphilones and isolated β-carotene, have already found commercial and industrial applications, and most of the fungi explored for the production of pigments are from a few genera such as *Monascus*, *Talaromyces*, *Aspergillus*, *Penicillium* and *Fusarium*. Lagashetti et al. (2022) focused on a less-common fungal species, isolated from the infected leaves of *Maytenus rothiana* and identified via morphological and molecular methods as *Gonatophragmium triuniae* [13]. Its growth under different conditions revealed the conditions suitable for pigment production, and biological screening demonstrated the antibacterial and antioxidant activities of *G. triuniae* extracts. The major orange-colored pigment produced by the species was identified as 1,2-dimethoxy-3 H-phenoxazin-3-one. The authors note that pigments and other bioactive secondary metabolites of *G. triuniae* have potential applications in the textile and pharmaceutical industries. 

Fungi of the genus *Penicillium* are widely studied as sources of bioactive compounds, but research on these fungi and their metabolites is far from complete, as shown in the work of Cadelis et al. (2022) [14]. Studying P. *bissettii* and *P*. *glabrum*, this group isolated five known polyketide metabolites, penicillic acid, citromycetin, penialdin A, penialdin F and myxotrichin B. During the derivatization of penicillic acid, a novel dihydro derivative was produced, providing evidence for the existence of an open-chained γ-keto acid tautomer in the starting material. Penicillic acid and penialdin F were found to inhibit the growth of methicillin-resistant *S. aureus*, which is important in view of the clinical problems associated with resistant microbial strains, which mainly involve hospitalized patients. Two other metabolites, penialdin F and citromycetin, were active against *Mycobacterium abscessus* and *M. marinum* [14].

This Special Issue also received three interesting review contributions. The first of these addressed recently discovered secondary metabolites from *Streptomyces* species and was prepared by Lacey and Rutledge [15]. Their review, with a particular focus on the year 2020, presented 74 novel secondary metabolites from *Streptomyces* species, with a wide range of chemical scaffold variability, including the cyclic peptides ulleungamide, viennamycins A and B and pentaminomycins C–E, metabolites with complex chemical structures. Linear peptides such as spongiicolazolicins A and B were isolated from marine species, and linear polyketides (e.g., adipostatins E–J, trichostatic acid B, trichostatin A and chresdihydrochalcone), terpenoids (e.g., napyradiomycins and flaviogeranins), polyaromatics (e.g., baikalomycins A–C and gardenomycins A and B), macrocycles (e.g., conglobatins and somamycins) and furans (e.g., furamycins) are among the compounds discussed in the review [16]. The authors noted that the *Streptomyces* species reported in their review were isolated from a wide range of environments, possess a diversity of novel chemical structures and represent a thriving and multifaceted area of drug discovery research.

The second review contribution focused on ^13^C-NMR data from 504 pentacyclic triterpenoids isolated from plants of the Celastraceae family, covering the period of 2001 to 2021 [15]. This class of secondary metabolites is of the utmost importance as they are reported to possess varied biological potential as antiviral, antimicrobial, analgesic, anti-inflammatory and cytotoxic agents against various tumor cell lines. The review covered the pentacyclic triterpenoids of friedelane, quinonemethide, aromatic, dimer, lupane, oleanane and ursane, among other classes. The data reported by Camargo et al. (2022) highlighted the amazing structural diversity of pentacyclic triterpenes and the complexity of some representatives, such as the dimeric molecules [15]. The ^13^C-NMR data presented in this review are an enormous contribution to the structural elucidation of new compounds of this class of terpenes.

Last, but not least, Amen and colleagues from Prof. Shimizu’s group reviewed the sources, bioactivities, biosynthesis and spectroscopic features of naturally occurring chromone glycosides. The compounds addressed in the review were described from plants (angiosperms) of thirty-three families, three families of ferns, four species of lichens, three species of fungi and three families of actinobacteria [17]. O-glycosides or C-glycosides were analyzed separately; phenyl and isoprenyl chromone glycosides and phenyl ethyl chromone glycosides were then analyzed, followed by the class of chromone glycosides with additional heterocyclic moieties. Hybrids of chromones with other classes of secondary metabolites, hybrids of furano-chromones with cycloartane triterpenes and hybrids of chromones with secoiridoids were presented in sequence, and finally, representants of the groups of chromone alkaloids and aminoglycosides, were discussed. In the second part, the spectroscopic features of the chromones were carefully described, including UV, IR, ^1^H and ^13^C-NMR data of the 192 chromones [17]. This is indeed a great collaboration for the prompt identification of chromone metabolites and an incentive to conduct deeper studies with this class of metabolites.

In view of the papers that comprise this Special Issue, we believe that our objectives have been achieved. We are thankful for all the contributions received, and hope that the papers will be of interest to all readers of *Molecules*. Finally, natural products still have much to contribute to humanity, and we wish great results and good discoveries to all authors and readers in their upcoming research.

## References

[1] Jiao Y., Yang Y., Zhou L., Chen D., Lu Y. (2022). Two Natural Flavonoid Substituted Polysaccharides from *Tamarix chinensis*: Structural Characterization and Anticomplement Activities. Molecules.

[2] Ferreira J.d.F., López M.H.M., Gomes J.V.D., Martins D.H.N., Fagg C.W., Magalhães P.O., Davies N.W., Silveira D., Fonseca-Bazzo Y.M. (2022). Seasonal Chemical Evaluation of *Miconia chamissois* Naudin from Brazilian Savanna. Molecules.

[3] Teixeira A.F., de Souza J., Dophine D.D., de Souza Filho J.D., Saúde-Guimarães D.A. (2022). Chemical Analysis of *Eruca sativa* Ethanolic Extract and Its Effects on Hyperuricaemia. Molecules.

[4] da Silva G.G., Pimenta L.P.S., Melo J.O.F., Mendonça H.d.O.P., Augusti R., Takahashi J.A. (2022). Phytochemicals of avocado residues as potential acetylcholinesterase inhibitors, antioxidants, and neuroprotective agents. Molecules.

[5] da Luz J.R.D., Barbosa E.A., Nascimento T.E.S.D., de Rezende A.A., Ururahy M.A.G., Brito A.d.S., Araujo-Silva G., López J.A., Almeida M.d.G. (2022). Chemical characterization of flowers and leaf extracts obtained from *Turnera subulata* and their immunomodulatory effect on LPS-activated RAW 264.7 macrophages. Molecules.

[6] Jung E.P., de Freitas B.P., Kunigami C.N., Moreira D.D.L., de Figueiredo N.G., Ribeiro L.D.O., Moreira R.F.A. (2022). *Bauhinia forficata* Link infusions: Chemical and bioactivity of volatile and non-volatile fractions. Molecules.

[7] Do L.T., Huynh T.T., Sichaem J. (2022). New Benzil and Isoflavone Derivatives with Cytotoxic and NO Production Inhibitory Activities from Placolobium vietnamens E. Molecules.

[8] Ware I., Franke K., Hussain H., Morgan I., Rennert R., Wessjohann L.A. (2022). Bioactive phenolic compounds from *Peperomia obtusifolia*. Molecules.

[9] Jimoh T.O., Costa B.C., Chansriniyom C., Chaotham C., Chanvorachote P., Rojsitthisak P., Likhitwitayawuid K., Sritularak B. (2022). Three new dihydrophenanthrene derivatives from *Cymbidium ensifolium* and their cytotoxicity against cancer cells. Molecules.

[10] Thant M.T., Khine H.E.E., Nealiga J.Q.L., Chatsumpun N., Chaotham C., Sritularak B., Likhitwitayawuid K. (2022). α-Glucosidase inhibitory activity and anti-adipogenic effect of compounds from Dendrobium delacourii. Molecules.

[11] Barakat H., Qureshi K.A., Alsohim A.S., Rehan M. (2022). The Purified Siderophore from *Streptomyces tricolor* HM10 Accelerates Recovery from Iron-Deficiency-Induced Anemia in Rats. Molecules.

[12] Wei Y., Wang J., Liu Z., Pei J., Brennan C., Abd El-Aty A.M. (2022). Isolation and characterization of bacteriocin-producing *Lacticaseibacillus rhamnosus* XN2 from yak yoghurt and its bacteriocin. Molecules.

[13] Lagashetti A.C., Singh S.K., Dufossé L., Srivastava P., Singh P.N. (2022). Antioxidant, antibacterial and dyeing potential of crude pigment extract of *Gonatophragmium triuniae* and its chemical characterization. Molecules.

[14] Cadelis M.M., Nipper N.S., Grey A., Geese S., van de Pas S.J., Weir B.S., Copp B.R., Wiles S. (2021). Antimicrobial Polyketide Metabolites from *Penicillium bissettii* and *P. glabrum*. Molecules.

[15] Lacey H.J., Rutledge P.J. (2022). Recently discovered secondary metabolites from *Streptomyces* species. Molecules.

[16] Camargo K.C., de Aguilar M.G., Moraes A.R.A., de Castro R.G., Szczerbowski D., Miguel E.L.M., Oliveira L.R., Sousa G.F., Vidal D.M., Duarte L.P. (2022). Pentacyclic Triterpenoids Isolated from Celastraceae: A Focus in the ^13^C-NMR Data. Molecules.

[17] Amen Y., Elsbaey M., Othman A., Sallam M., Shimizu K. (2021). Naturally occurring chromone glycosides: Sources, bioactivities, and spectroscopic features. Molecules.

